# Transference interventions in psychodynamic psychotherapy for adolescent depression: a qualitative study

**DOI:** 10.3389/frcha.2026.1789526

**Published:** 2026-05-08

**Authors:** Rune Johansen, Hanne-Sofie Johnsen Dahl, Anna Grasdal Lunde, Rebekka Thorkildsen Sem, Mathilde Sem Thorkildsen, Sarah Christine Fahs, Randi Ulberg

**Affiliations:** 1 Division of Mental Health and Substance Abuse, Diakonhjemmet Hospital, Oslo, Norway; 2 Department of Psychology, Faculty of Social Sciences, University of Oslo, Oslo, Norway; 3 Division of Mental Health and Addiction, Institute of Clinical Medicine, University of Oslo, Oslo, Norway; 4 Department of Child- and Adolescent Psychiatry, University of Oslo, Oslo, Norway; 5 Faculty of Health Sciences, VID Specialized University, Oslo, Norway; 6 Research Unit, Division of Mental Health, Vestfold Hospital Trust, Tønsberg, Norway; 7 Child and Adolescent Mental Health Research Unit, Division of Mental Health and Addiction, Oslo University Hospital, Oslo, Norway

**Keywords:** adolescent psychotherapy, depression, psychoanalytic, psychodynamic, transference

## Abstract

**Introduction:**

Psychodynamic therapy is based on the understanding that past experiences, relationships, and unconscious conflicts can influence a person's current behavior and mental state. Transference is a central construct in psychodynamic psychotherapy, which refers to the emotions and attitudes that the patient attributes to the therapist. These emotions shape the patient's perception of both the therapist and the therapeutic process. Emphasizing and exploring how transferential phenomena manifest within the therapeutic relationship is assumed to have a beneficial therapeutic effect. The primary aim of the present qualitative study was to identify examples of such transference interventions in short-term psychoanalytic psychotherapy with adolescents diagnosed with depression.

**Method:**

Examples of transference interventions were identified from randomly selected sessions, as well as from two therapies with good outcomes.

**Results:**

Transference interventions were used to a moderate extent and were primarily aimed at encouraging patients to reveal and discuss their thoughts and feelings about the therapist and the therapy.

**Discussion:**

Transference interventions in psychotherapy with adolescents with depression seemed mostly to be preparatory and directly relevant to the young person's present, everyday life. The interventions were often posed as open question.

## 1 Introduction

Psychodynamic therapy recognizes that a person’s present behavior and mental state can be shaped by past experiences, relationships, emotions, and unconscious conflicts.

Short-term psychodynamic psychotherapy (STPP) is a time-limited treatment built on general psychodynamic principles. The manualized STPP described by Cregeen and colleagues (1) consists of 45-min weekly sessions for 28 weeks. In STPP, the therapeutic relationship provides the framework for both the patient–therapist dialog and the therapist's interventions. The overall aim of the therapy is to alleviate depressive symptoms, help the young person through the developmental phase in relation to peers and parents, prepare for the transition to the workforce, and prevent psychiatric problems in adulthood. In STPP, the therapist's interventions are intended to guide the adolescent through an uncovering process, in which more of the young person's unconscious motives and fantasies are revealed.

In the United Kingdom-based RCT entitled IMPACT (Improving Mood with Psychoanalytic and Cognitive Therapies (2), STPP was implemented as a treatment modality for adolescents diagnosed with depression. STPP was compared with cognitive behavioral therapy and a brief psychosocial intervention, and was found to be as effective as the two other treatment modalities.

Transference, free association, emotions, and the unconscious are core concepts in psychoanalysis and psychoanalytic psychotherapy (PDT). Transference interventions (TIs)—including interpretation of avoidance, defense mechanisms, and resistance—are central to exploring deeper layers of the psyche and are thought to facilitate improved insight, as well as symptomatic, relational, and functional improvement. The concept of transference was introduced by Sigmund Freud, who also described it in the treatment of an adolescent (3, 4).

Throughout the history of PDT, up to the present day, the importance of transference interventions has been maintained (1). The development of transference interventions as a valuable concept, along with empirical findings of interventions across various personality pathologies, has been summarized in the literature (5, 6). These concepts are also established in STPP, but with a somewhat different focus. In Malan’s (7) original work on short-term psychotherapy, he sought to systemize psychodynamic theory into two “triangles.” The triangle of conflict has three corners: One represents feelings or impulses, another represents anxiety, and the third represents psychological defenses. Symptoms and maladaptive patterns are understood as defensive solutions to manage anxiety about underlying feelings. The triangle of persons explains where these conflicts emerge in relations: One corner represents important past relationships, the second represents current relationships, and the third represents the therapeutic relationship or transference. Kernberg (5) developed and empirically tested a treatment model for more severe personality disorders. Named Transference Focused Therapy, it is a highly structured, twice-weekly psychodynamic therapy that focuses almost exclusively on transference and the patient's moment-to-moment experience of the therapist (5).

The aim of transference interventions has changed throughout the history of psychoanalytic and psychodynamic therapy, and there now appears to be a greater consensus on grounding these interventions in the here-and-now therapeutic process. Definitions of the concept of transference have included a broad variation in what constitutes transference interventions. In updated work on personality pathologies, the focus in short-term therapies is largely the same as in long-term therapies. The main difference lies in the shorter time and more focused goals, with an emphasis on working with transference interpretations (8). TIs can be defined as a specific treatment technique believed to initiate a chain of events leading to insight and maturational dynamic change. Transference work (TW) refers to such transference interventions and the subsequent interaction between therapist and patient, and involves analysis of the patient–therapist relationship. TW is considered to be one of the key ingredients in PDT (4, 9, 10).

However, there is a lack of empirical data on the effect of transference interventions in PDT in adolescent populations. Establishing timely and effective treatment approaches for adolescent depression is therefore considered an important undertaking (9).

The First Experimental Study of Transference Work-In Teenagers (FEST-IT)—a randomized controlled trial of psychotherapy conducted for adolescents diagnosed with depression in Norway—demonstrated that a moderate application of TIs yielded particularly strong benefits (11). In a recent review of the evidence base for psychosocial and combination treatments for child and adolescent depression, the IMPACT and FEST-IT studies were highlighted as core contributions (30).

To explore TW in the present nested qualitative study, we used the five categories of transference interventions described in the First Experimental Study on Transference Interpretations (4, 12, 13), a study conducted on adults, with examples of transference interventions from time-limited (40 weekly sessions) PDT with adults.

### 1.1 Categories of TI

(1) The therapist addresses transactions in the patient–therapist relationship (address transaction).
(2) The therapist encourages exploration of thoughts and feelings about the therapy and the therapist's style and behavior (thoughts and feelings about therapy).
(3) The therapist encourages patients to discuss how they believe the therapist might feel or think about them (beliefs about therapist).
(4) The therapist includes himself/herself explicitly in interpretive linking of dynamic elements (conflicts), direct manifestations of transference, and allusions to the transference (linking therapist to dynamic).
(5) The therapist interprets repetitive interpersonal patterns (including genetic interpretations) and links these patterns to transactions between the patient and the therapist (repetitive interpersonal pattern).

Categories 1–3 may be considered preparatory TIs, primarily involving clarification and confrontation (6). These TIs aim to emphasize and explore the interaction in the therapy room and increase the patient's awareness of the therapeutic relationship. Categories 4–5 are considered proper transference interpretations, with Category 5 including genetic interpretations.

Drawing on data from the IMPACT study (“Improving mood with psychoanalytic and cognitive therapies” (2), Della Rosa and colleagues conducted a qualitative study of four adolescents patients with depression, each seen for four sessions, focusing on their responses to the use of TIs directed at the ending of the therapy. They identified two main types of response to this transference work, which they classified as “dramatizing” and “downplaying” responses. They concluded that “it may be worth considering whether transference interpretations linked to endings are more appropriate with certain kind of adolescent patients than others, and whether it is important to maintain a flexible approach to the exploration of transference in relation to endings and separations when working with adolescents” (14).

From the same study, Cirasola and colleagues (15) examined how TW affected alliance ruptures. They examined 16 sessions from four adolescent therapies and reported that TW was commonly employed and most frequently targeted immediate issues within the therapeutic relationship, rather than linking them to external relationships. Overall, this form of TW made a positive, albeit modest, contribution to the repair of alliance ruptures.

To the best of our knowledge, apart from these two papers from the IMPACT study, there are no other studies reporting on the use of TIs and their impact on in-session processes and long-term outcome in adolescent psychotherapy. Therefore, further research is needed to deepen the understanding of how TIs are applied in adolescent psychotherapy and to examine both their immediate and long-term effects.

### 1.2 Background of the current study

In the present study, data were drawn from FEST-IT, a randomized controlled trial investigating the effects of transference interventions in STPP for adolescents with depression. The study design has been described in greater detail by Ulberg et al. (11, 16).

#### 1.2.1 Aim

The main aim of the RCT was to investigate the effects of transference interventions in adolescents diagnosed with major depressive disorder.

#### 1.2.2 Method

The participants were 70 adolescents with an ongoing moderate to severe unipolar depression, diagnosed according to the Diagnostic and Statistical Manual of Mental Disorders, Fourth Edition (DSM-IV; 31). The participants were of both sexes and aged 16–18 years. Comorbidity was expected to be common among adolescents and was not an exclusion criterion. Adolescents with developmental disorders, learning disabilities, drug addiction and psychotic disorders were excluded.

Patients were referred to outpatient clinics in the southeastern region of Norway. A total of 70 adolescents were included in the study. At the start of the treatment, the two randomized groups were similar. The mean age was 17.5 years, with the majority being girls (82%). 42% of participants lived with both parents, while 49% resided with one parent or commuted between parents. All participants fulfilled criteria for severe depression, and 8% had a diagnosed personality disorder, as determined by a semistructured interview. Patients in both groups received STPP. One group received therapy with a moderate degree of TIs, while the other received therapy without TI (16). The therapy involved 28 weekly sessions of 45 min each. Assessments were conducted at pretreatment, during treatment, post-treatment, and 1-year follow-up.

Given that STPP with adolescents requires therapists to be experienced in treatment of children and adolescents, all therapists were specialists in psychiatry or clinical psychology with at least 2 years of training in psychodynamic psychotherapy. Before inclusion of participants, the therapists completed a 1-year course in psychodynamic psychotherapy, both with and without the use of TIs. Therapists treated patients in both treatment groups. Randomization was blind to evaluators and researchers; only the therapists knew whether the patients were randomized to the transference group or the non-transference group.

The level of depressive symptoms was assessed by clinicians using the Montgomery–Åsberg Rating Scale (MADRS) (17). Patients also completed the Beck Depression Inventory (BDI) (18). Both measures were administered at pretreatment, after session 12 and 20, at post-treatment (session 28), and at the 1-year follow-up.

Assessing symptoms related to diagnoses alone does not necessarily provide a comprehensive picture of the effects of therapy. Therefore, the Psychodynamic Function Scale (PFS) was also used (score range 0–100, with scores above 70 indicating good functioning) based on a semistructured interview focusing on relational and intrapsychic functioning. The PFS provides information about how patients manage their relationships with others, solve problems, and understand their own functioning, rather than focusing solely on symptoms. The PFS has been shown to be useful in therapy with adolescents (19, 20). PFS scores were obtained before treatment, after treatment, and at the 1-year follow-up.

The Transference Work Scale (TWS) is a rating tool designed to categorize and analyze transference interventions. The tool is considered particularly useful for evaluating transference work in studies combining quantitative and narrative data. The TWS consists of 26 items divided into seven subscales. These subscales aim to identify, categorize, and assess the timing, content, and valence of the TI. Finally, the patient's response to the transference work is also rated. The items are scored on a Likert scale from 0 to 4, where 0 represents “not at all” and 4 represents “very much” (13, 21). TIs are categorized on the basis of five TI categories and the TWS items are scored either “Yes” or “No.” It has been reported that interrater reliability was good to excellent for most of the 26 questions in the TWS items (13).

#### 1.2.3 Results

The main findings of the study were that transfer interventions had a positive effect on treatment outcomes at the 1-year follow-up (Cohen's *d* = 0.53 for BDI, *d* = 0.69 for MADRS). Adolescents in the non-transfer group experienced slightly faster improvement up to the 12th session. From the 12th session to the 1-year follow-up, their improvement remained stable. In contrast, patients in the transfer group continued to improve, showing further reductions in depressive symptoms up to the 1-year follow-up assessment (11).

The participants were comprehensively and reliably assessed for symptoms consistent with personality disorders (22). A small but notable additional effect of transference interventions was found in the transference group among adolescents with personality pathology (including emotionally unstable personality disorder). Patients with a higher number of personality disorder criteria at the start of treatment showed a better treatment effect, and the difference between the groups persisted up to 1 year after treatment termination. Adolescents with severe depression and comorbid cluster B personality symptoms appeared to benefit more from STPP with transference interventions than adolescents without such personality pathology (23). Participants forged healthier relationships with family and peers, cultivated a more optimistic future outlook, and reported enhanced self-perception and relational expectations—factors that appear pivotal for continued development (24). Patients who discontinued treatment early displayed heterogeneous, multidimensional dropout reasons, implicating factors such as therapeutic effort, session structure, and the quality of therapist–patient communication (25).

## 2 Aim

The aim of the present qualitative study was to identify examples of TIs in STPP with adolescents diagnosed with depression. In particular, we sought to investigate the use of TIs over the course of treatment and to analyze the patient–therapist dialog following these interventions. Examples of the five different categories of TIs are presented from clinical work with adolescents in the study, followed by extended details of the effects of transference work in two therapies.

## 3 Method

### 3.1 Identification of examples of TIs, therapist use, and patient responses to TIs

Two experienced psychodynamic therapists listened to 20 randomly selected audio-recorded sessions 12 from every third patient in FEST-IT. They rated the use of TIs with TWS scoring on a 5-point Likert scale. Interrater agreement was calculated in SPSS. TIs were identified, transcribed, and categorized by RTS under the supervision of RU.

In addition, two good-outcome cases randomized to transference therapy and completing the full course of therapy (28 weeks) were selected to exemplify the use of TI throughout therapy. These cases were chosen based on the absence of a diagnosable major depressive disorder at the 1-year follow-up and low scores on depression scales: BDI (1 and 3) and MADRS (2 and 4). Sessions 3, 12, 20, and 28 from each therapy were analyzed to identify TIs and patient responses, as these time points coincided with the administration of quantitative measures. Material from these sessions was transcribed and rated with the TWS to identify, categorize, and evaluate transfer work in therapy. Two medical students (AL and MT) were trained in TWS scoring by Randi Ulberg (13). The students rated the transcripts individually.

The two students also listened to all therapy sessions in both treatments to obtain a comprehensive picture of patient histories and treatment courses. This was important for assessing whether there were circumstances in the patients’ lives, or systematic differences in the use of TI, that were not captured in the TWS-rated sessions (26).

## 4 Results

TIs were identified in 15 of the 20 audio-recorded sessions. The level of TIs was moderate in the transference group and low in the non-transference group (Table 1).

**Table 1:** Level of transference interventions in therapy with adolescents receiving 28 weeks of psychoanalytical psychotherapy with or without transference interventions (11, 27).

Outcome measures	Transference group			Non-transference group			Df	*t*	*p*
	*N*	Mean	(SD)	*N*	Mean	(SD)			
The present study (*N* = 20)	15	2.47	1	5	0.2	0.3	18	7.7	0.000
In the whole study (*N* = 69)	39	2.2	1.47	30	0.52	0.78	52.5	5.5	0.00

**Table 2:** Baseline outcome measures in 15 adolescents receiving 28 weeks of psychoanalytical psychotherapy with transference work.

Outcome measures	Mean	Minimum	Maximum	SD
BDI (Becks Depression Inventory)	29.1	11.0	46.0	10.2
MADRS (Montgomery and Åsberg Depression Rating Scale)	22.1	9.0	33.0	6.4
GAF (Global Assessment of Functioning)	59.2	50.5	67.7	4.9
PFS (Psychodynamic Functioning Scales)	60.9	55.7	70.4	3.8

Intra-class correlation between the two raters (single measure) across the whole sample was.89 (95% CI 0.75 to 0.96, *p* = 0.000) (11, 27).

In the following, we will present examples of TIs from these randomly selected sessions to illustrate the five different TI categories.

### 4.1 Examples of TI in adolescent PDT

#### 4.1.1 Category 1: the therapist points to the patient–therapist relation

T: How are you?
P: I'm not doing well, but I don't like talking about it. I don't know what to say.
T: But you’ve come to our session today. Do you want to talk about it here?
P: I could, but I really don't want to. That’s why I cancelled our previous session.
T: But you chose to come today…
P: Yes, but I didn’t want to.
T: That's the point. Even though it's difficult, you can talk about what's hard here.

In example 1, the therapist simply points toward the two of them sitting together in the room and adds a question that encourages the patient to reflect on the therapeutic relationship. The question reveals negative and difficult feelings associated with the therapy. As noted in the introduction, from a different point of view, some might not categorize this intervention as a TI, but rather as a clarification or invitation to a later TI. In this study, however, we categorize it as a TI based on the patient's emotional experience of the relationship.

#### 4.1.2 Category 2: exploration of the patient’s thoughts about the therapy/therapist

P: … I don't think it helps to talk about the things I don’t want to talk about.
T: What were your initial thoughts on starting therapy? Were you hesitant?
P: No, I knew what therapy was… Mom thought it would be good for me, but she let me decide. She said I could go if I wanted to.
T: Have things changed since you started coming here?
P: I don't think things have changed too much. I was hoping to get better, and either stop being sad or stop crying during each session.
T: Why do you see crying as something bad?
P: I don't. But when I cry, I know it means I'm sad. And I don't want to be sad.
T: So, you don't feel sad outside our sessions?
P: Well, I do. I always end up crying when I argue with someone. When I first started coming here, I was hoping I would cry it all out in here, so I could cry less elsewhere.
T: So, you also feel sad outside our sessions?
P: Yes.
T: Last week, you told me you were doing well. Now you say you sometimes feel sad.
P: Yes, I've been sadder lately… I've been thinking about my friends…
T: Yes, I understand something came up…
P: … And about Dad. We don't spend time together as he promised.
T: No.
P: In the past week I've been home late, because my driving lessons finish at 8 pm. I've been looking forward to coming home and telling him about our sessions… He said he wanted to hear about them. But when I get home, he doesn't have the time. And then I have to go to bed… We don't argue anymore… But we don't talk as he promised.
T: Yes, I understand. Are you disappointed?
P: Yes.

This example presents three Category 2 interventions. Through the interaction, the therapist continues to encourage the patient to explore painful themes. The therapist also encourages the patient to apply, outside of therapy, the emotional experiences and insight acquired within therapy. Here, the therapy (or therapist) becomes a more central part of the TI, shifting focus from a more general relational experience to a therapeutic relational perspective.

#### 4.1.3 Category 3: what the patient thinks about the therapist

P: I can't talk when I cry…
T: In that case, I can understand why it's hard for you to cry during our sessions. Perhaps you're worried I might see you as weak?
P: Mmm…
T: It seems to me that you're getting in touch with some emotions?
P: What do you mean?
T: It seems your emotions are stirring something important in you… Something connected to your thoughts.
P: Mmm…
T: …We talked about your birthday… And the emotions you couldn't get rid of… It seems you try to push your emotions away, because crying makes you feel vulnerable? Perhaps you're scared I would perceive you as weak?
P: I don't know, but it feels uncomfortable.
T: Yes, it is uncomfortable.

The therapist opens with an unusual intervention and follows it with three more interventions. Each time, the therapist becomes increasingly direct and confrontational toward the patient. These interventions are direct invitations to explore the patient's experiences of the therapist, and are therefore classified as category 3 TIs. However, the patient continues to avoid exploring what the patient believes the therapist thinks about the patient.

#### 4.1.4 Category 4: the therapist is included directly in the intervention

P: It’s hard not to think about it… People at school keep bringing it up.
T: Do you feel like I've pushed you into thinking about it, just like everyone at school? Would you rather not think about it?
P: Not directly. But you've asked questions that have reminded me of it. I couldn't answer your questions without thinking about it. It triggers itself, I guess. It's strange.
T: Mmm…

In the material we identified few category 4 interventions. In this example, the therapist intervenes by making a direct connection to the patient's experience of the therapist, and the patient responds to the interventions by further exploring what the therapist has pointed to. This is a clear example of a category 4 TIs.

#### 4.1.5 Category 5: interpretations of repetitive interpersonal patterns appearing in the therapy

T: It sounds like you get angry with him?
P Yes, or I get upset… and frustrated.
T: Mm…
P: He doesn't notice all the things I do throughout the day.
T: Last week, you said your relationship with your parents would be better if you didn't live together. You seemed to get angry with me because you felt I didn't understand you. The same way you get upset with your father when he doesn't understand you?
P: It wasn't directed at you personally. I just feel so lonely. There's so much going on in my head. People say I overreact and call me a *drama queen*. Am I too sensitive? Is *that* the problem?

Here, the therapist directly interprets repetitive interpersonal patterns that appear both outside the therapy room and in the here-and-now towards the therapist. In our classification, this is an example of a category 5 TIs.

### 4.2 Illustrating the application of TIs during the course of therapy

We now present two vignettes to illustrate a more detailed and thorough use of TIs during the course of therapy.

#### 4.2.1 Patient Anna

Anna is a 16-year-old girl who lives in Oslo, the capital of Norway. She lives with her mother, father, brother, and their dog. As a child, she moved from the southern part of Norway to Oslo. At the time of treatment, Anna was in a transitional phase from secondary school to upper secondary school and was unhappy, especially when at school. She found it difficult to connect with peers and had few friends. During these difficult times, she struggled with self-harm and isolation. She had a MADRS score of 28 and a self-reported BDI-II score of 33. On the PFS, she was rated 47.5, which indicates serious interpersonal struggles. According to DSM IV criteria, she was diagnosed with major depressive disorder and was rated with a high level of suicidality. She also met criteria for an unspecified personality disorder (14 criteria; e.g., schizoid and avoidant traits).

##### 4.2.1.1 Examples of TIs in the context of therapy with Anna

In the two initial therapy sessions, Anna appeared deeply depressed. She had recently become aware that a close family member was seriously ill and might pass away in the near future. Emotions related to this sad news were explored. The focus was also on how to take care of herself in this stressful situation.

##### 4.2.1.2 Session 3

*In this session, the focus was on self-harm and Anna’s feeling of being kept under observation by her parents*. T: What do you think about coming here to talk to me about how you feel and what happens at home?
P: Right now, I don't really care much about talking. Besides, I don't feel any different from us talking about the situation.
T: Since we don't have much time left today, we might continue this topic in the next session. Do you have any thoughts about what you want us to work on during therapy? Is there something that makes sense to you?
P: No, not really. To be completely honest, I don't know.
T: The purpose of therapy is that it should be meaningful for you. I've mentioned to you previously that maybe your relationships with other people will be the main subject?
P: Mmm …

The therapist wants to explore the patient's thoughts and feelings about the therapy with an initial TI, a Category 2 intervention. Anna's first response is negative or perhaps ambivalent. The therapist then tries to strengthen the alliance while offering another, weaker Category 2 intervention. It would be interesting to explore the therapist's countertransference, given how generalized this intervention is.

Between sessions three and twelve, much of the conversation is about school and the theoretical driving test she needs to pass, which she dreads. She spends a lot of time at home and does not go to school regularly, but eventually adapts to everyday life at a new school. During this period, the patient struggles with significant inner tension, self-harm, and poor self-image, and she is absent from several therapy sessions.

##### 4.2.1.3 Session 12

In this session, the focus is on Anna’s relationship with her parents, the fact that she has missed several therapy sessions, her self-harm, and her suicidal thoughts.

T: It is nice to see you again. It's been a while since you were here. Even though it is voluntary to come, it seems to have been difficult for you to make it happen. Coming here is something you do for yourself. Did you get the reminder on your phone yesterday?
P: Yes, I did.
T: Mhm… I just wondered—since reminders were also sent the two times you did not come. What did you think I was thinking when you did not come?
P: Mm, no, I felt I should say that I wouldn't come. I just did not. I do not know what you were thinking, really.
T: Mm… No, you could not know what I was thinking. But what did you think I was thinking?
P: Yes, what did you think…? I thought maybe you were thinking that I was such a typically lazy teenager.
T: (Laughs) Do you think I thought so?
P: No, I don't know.
T: No, seriously. What did you think I might think about you?
P: I did not think anything like that in particular, really. I thought you might have been disappointed. Maybe not exactly disappointed… I do not quite know how to put it into words. I actually don't know what you were thinking.
T: Mm… I have no reason to be disappointed; this is my job.
P: Maybe I'm turning the disappointment toward myself.
T: I would like you to come, without any expectations. I in fact wondered if you might have felt so ill that you could not come, or if something kept you from coming, or if it was difficult for you to cancel our appointment.

TIs from two different categories are used. The initial TI is a mix between Category 1 and 2 interventions, pointing to the interaction between therapist and patient. In the following transference work, the therapist repeatedly asks what the patient thinks the therapist thinks and feels about her. It seems difficult for the therapist to address the relationship from their own side, but still, this can be regarded as a Category 3 intervention.

Between sessions 12 and 20, the patient changes her social attitude at school. She talks more with classmates, feels much better, and jokes and laughs a lot in the sessions. Difficult days with more depressive thoughts are less common and she has started exercising. The patient is able to find ways to cope with difficult feelings. Still, there are some sessions she does not show up to.

##### 4.2.1.4 Session 20

In session 20, the therapist and patient talk about practical solutions for organizing the upcoming sessions, so that it will be as easy as possible for her to attend while also avoiding absence from school.

T: These days, when you feel down, it doesn't seem to last as long. It seems like you manage to recover more quickly?
P: No, well, I've noticed that it helps me a lot when people say hello, for example. It also helps that I dare to talk to other pupils at school.
T: Yes, you dare to greet others!
P: You are proud of me, right? Yes (laughs a lot).
T: (Laughs) Proud of you. Yes, yes, that's great! Did you notice that I'm a little proud of you?
P: Mm, yes, I think you seem happy, so yes. At least am I very happy!
T: You seem gentler and happier.
P: Mmm, yes.

Here, it is the patient who takes the initiative to comment om relational matters, first in relation to people in general, then to classmates, and finally the therapist. The therapist confirms the relational aspect between them, and we classify this as a Category 3 TI.

In the sessions between sessions 20 and 27, the patient states that she is feeling better. She says that she has a boyfriend, feels comfortable at the new school, and includes the therapist in her thoughts about the future.

##### 4.2.1.5 Session 28

In this session, the focus is on the fact that this is the last one. Anna and the therapist talk about the course of therapy and about her experience of attending therapy. Anna also talks about her hopes for the future, her wishes regarding school and education, and future career paths.

T: I also think it's a bit moving that this is your last session
P: That’s nice to hear (laughs).
T: It has been very nice to get to know you!
P: Likewise! Well, I don't actually know you, but I kind of know who you are (laughs)! I know that psychologists should be a little anonymous, in a way. They are not supposed to tell very much about themselves.
T: When we first met, you struggled a lot. You were tormented by depressive and suicidal thoughts. I think it's nice to see that you've become a lot better.
P: Yes, my whole family says the same. That's the best thing after being here in therapy. Next year the two of us may celebrate. Then I'm 18 and then we can go partying!
T: (Laughs) Oh, is that what you want?
P: No, I'm just kidding you. I just wanted to check how you reacted! I think you reacted in a good way.
T: Did you think that my reaction was a positive reaction?
P: Yes, yes! You will probably also be a good therapist for other patients. I have noticed that there are very many who feel the same way as I did. I just was not aware of that. I feel that's a lesson learned for me. When I recovered from the depression, I learned that many others have the same kind of troubles.
T: What have you learned?
P: Now, I am able to imagine how other people feel. I can put myself in someone else's place; how terrible they might feel.
T: It seems as if you are able to look at yourself from the outside and also identify with how other people feel.
P: Yes. I have learned that through the therapy process I have been through.
T: What is the most important thing you have learned here?
P: I have learned a lot. The most important thing is that I have learned that people should not be alone. Even though I sometimes think it's wonderful to be alone, I should not isolate myself but stick to my flock.

This extract shows different TIs, more from the patient than from the therapist. The first TI is used to introduce a new topic of conversation and is considered mediocre in terms of timing, context and in how precise and striking it is. Still, it is a Category 1 TI, as it points to the interaction between therapist and patient. Then, as in session 20, the patient takes the initiative to comment on the therapist—patient interaction. The therapist confirms the relational aspect, and we classify this as a Category 3 TI. The last TI aims to explore the patient's thoughts and feelings related to the therapist's reactions and is a Category 2 intervention.

At post-treatment, Anna had an average PFS score of 73.2, MADRS of 6, and BDI-II of 0. One year after treatment, her PFS was 69.6, MADRS was 6, and BDI-II was 2.

#### 4.2.2 Patient Betty

Betty is an 18-year-old girl attending upper secondary school in a non-urban area. Her parents are divorced. At the start of therapy, she lived with her father. She described a strained relationship with her mother. In addition to this, she had a long history of difficult relationships with peers. Before treatment, she was measured with a MADRS score of 14, but rated herself with a BDI-II score of 31. On PFS, she had a score of 65. According to DSM IV, she was diagnosed with recurrent depression, major depressive disorder, panic disorder, agoraphobia, and social phobia.

Initially, therapy focused on Betty's depressive symptoms and the associated loss of functioning. In the first two therapy sessions, Betty focused on her relationship with her parents, difficulties with friendships, and issues related to sexuality.

##### 4.2.2.1 Examples of TIs in the context of therapy with betty

###### 4.2.2.1.1 Session 3

In this session, the patient’s relationship to food, as well as the relationship with her parents, is the main focus of the conversation.

P: I don't know if I really have that much more to say. I don't have much on my mind.
T: It sounds as if you don't want to talk but are wrapping up the conversation.
P: Well, I have made sure to be on time.
T: Yes, you're a little concerned about that.
P: I am very conscientious. I've really tried to fill the time, however, because I do not know what to say…
T: Yes exactly. So, there is something you have thought about here today?
P: I wonder if I have lactose intolerance. I just have to accept that I cannot stand eating specific things. I'd rather eat what I can. I know this, but it's hard talking about it without my parents getting annoyed.
T: Mm, but you said that you don't know what to talk about here. Does that mean that you thought about what to say during the session? You wanted to talk about your possible lactose intolerance.
P: I don't know why I'm getting that reaction. I want to eat the food, so I do not understand why I react then? It could be that I am scared and think I am allergic, so that it also triggers my body?
T: I meant what you said about not knowing what to talk about here.
P: Yes, it’s a little difficult to come up with things.
T: Is that how you feel?
P: Yes, at least with the nurse at school. She used to ask me some questions and stuff. It made me think a little about things.
T: Yes, so here you feel a little left to yourself?
P: Yes, maybe to a greater extent. But everyone works differently. I think I have to get used to it, because I spend some time getting to know people. Now, I feel much more comfortable here in the sessions with you than I did in the beginning. I guess I just have to get used to it.
T: We can explore this further in our next session.

Several Category 2 TIs are offered. The therapist encourages the patient to explore thoughts and feelings related to the therapy and the therapist.

Between session 3 and 12, the therapist and Betty explore her relationship with her mother and father. In session eight, it emerges that a family member has recently passed away. At the same time, there is an ongoing conflict between her mother and father. She describes fewer depressive symptoms. Difficult topics such as identity and fear of the future are discussed. The patient does not show up for session 9 and 11.

###### 4.2.2.1.2 Session 12

The focus of the session is on the patient's relationship with her parents. The conflict between them, and difficulties related to her housing situation are central themes.

T: It is difficult for you to balance between your parents.
P: Mhm, yes, I'm stuck in the middle. They're trying to say, “I'm right, right?” Then I say, “Yes and no.” However, that is not the answer they want. Now, I've told them that I do not want this kind of conversation anymore. I'm sick of it. It's a shame because it has affected the relationship between Mom and Dad. It has become clear that they are not such good friends.
T: How is that?
P: They have been somewhat too good friends. My theory is that they fill each other's emptiness. I do not think that it has been the best situation for them. They have been around each other too much.
T: I was thinking that it seems as if you are searching for a solution. You want to move out of home because it's better for you not being too close to your parents. After the previous therapy session, you forgot the next session. I'm wondering if that was related to ongoing conflicts and difficulties. In such a situation, I wonder if you feel it's better for you to distance yourself instead of coming here to talk with me.
P: I was glad I forgot to come here. I had so much on my mind and lots to do at school.
T: I also wonder if you actually were a bit mad at me.
P: No.
T. No?
P: No, I do not think so. I think I was just so sad. That was when I wanted acceptance and empathy. The worst thing about not showing up for the therapy session was that there was no one else I could talk to. I could not talk to Mom; I could not talk to Dad.
T: Yes, so you felt very alone after the last session? Here, you also felt alone?
P: Yes, that was a difficult day. I get sad when thinking about it now. I just hope it gets better in the future. I need distance from my parents, and they need to find themselves when I'm moving out. They have lived their lives through me. I really think it's good for everyone if we were apart for a while.

The transference work begins with an intervention of Category 4 where the therapist includes the patient—therapist relationship in the interpretation of the conflict outside therapy. The two subsequent TIs belong to Category 2. They aim to encourage the patient to explore her own feelings about the therapist and the therapy.

In the following sessions, Betty appears more depressed with suicidal thoughts. She describes increased anxiety and depressive symptoms during the last week. The pressure on Betty by the conflict between her mother and father, as well as the death of a family member, is explored.

The level of depression fluctuates during the next weeks. In session 19, the patient feels better.

###### 4.2.2.1.3 Session 20

The patient has returned to school again after the summer holidays and reports stress related to this.

P: For a long time I haven't told my parents anything about how I feel. I was afraid they would not take me seriously. They are just saying they are sorry and don't pay any real attention to my feelings. I almost never tell them about my struggles. Therefore, I can't expect to get attention.
T: I wonder if that's how feel here too. When you tell me something, you want or expect to feel relieved. But, have you not experienced getting a helpful response from me?
P: I feel like I do a lot of work myself. Here, I will tell you things and you might ask some questions. That helps in organizing my thoughts and feelings. After the therapy sessions, I think more about what we have been talking about, and then I find a solution that makes it better for me. Going repeatedly over it again is a very important part of the process to get better. I could not do this on my own. Therefore, I think it's helpful for me to come here. I'm getting more organized and structured. I get better help here than from my family because you are more neutral.

Betty talks about the relationship with her parents and that she experiences not getting enough attention from them. The therapist's TI explores the patient's expectations and thoughts about the therapy (Category 2).

In the sessions leading up to session 28, the patient gradually appears more depressed, but with some fluctuations. The patient's identity, her experience of lack of attention from her parents and the conflict between them, are discussed. In addition, her closest friends are less available. The end of therapy is soon approaching. The patient expresses her fear of ending therapy. After the time-limited therapy, she will have a new therapist in another outpatient clinic.

###### 4.2.2.1.4 Session 28

The patient appears more depressed. She reports many physical symptoms. The main focus is that this is the last session, and she reveals that she is tired of constantly changing therapists.

P: The thing is, I get really tired when changing therapist to the other outpatient clinic. There, I have to fill in a lot of forms. It is very demanding.
T: You also get really tired from coming here to talk too?
P: Yes, I do. When I must fill in forms here, I get more tired than when I do not. It also depends a bit on what we are talking about. With the new therapist, I am more tired than I am here. I get tired here too, but to a different degree. With the new therapist, I get so tired that I want to go home to bed afterward. That's why I think, “Am I going to get to school afterwards at all?” But now I have promised myself that I will go to her tomorrow, and then I will see what I will do. If I do not feel well enough afterwards, I will go home.

The therapist offers a TI that explores the patient’s feelings about the therapy (Category 2).

At post-treatment, Betty had an average PFS score of 79.6, MADRS of 22, and BDI-II of 21. One year after treatment her PFS score was 80, MADRS was 4, and BDI-II was 3.

## 5 Discussion

We have presented examples of different categories of TIs, as well as examples of their use in STPP with adolescents experiencing depression. Positive effects of TIs in adult psychotherapy have been documented in the research literature (6). Della Rosa and colleagues (14) discussed whether the use of TIs in adolescent psychotherapy is appropriate, suggesting that TW with adolescents should not be emphasized, as investing in the therapeutic relationship and exploring earlier relationships with parental figures—which is often the focus of transference work—may compete with adolescents’ developmental task of establishing their identity in relation to the peer group. Further, Cirasola and colleagues (15), drawing on the IMPACT study, reported that factors improving the effectiveness of TW included the therapist's ability to remain focused on transference dynamics in the here and now, as well as their capacity to affirm and flexibly adjust their use of TW techniques. In contrast, a rigid approach to TW and the fostering of dependency between the adolescent and the therapist or the therapy itself hindered the resolution of alliance ruptures.

The beneficial effects of psychoanalytic/psychodynamic psychotherapy with TIs for adolescents with depression are demonstrated in the RCT FEST-IT (11, 23). Moreover, neither the studies based on IMPACT (14, 15) nor the present study supports the view that TIs offered in STPP dwell on more theoretical and irrelevant relationship between the adolescent patient and the therapist or on past relationships. Rather, results from both RCTs exploring TIs in adolescents with depression show interventions that are directly relevant to the young person's present, everyday life.

Based on the definition of TI categories (1 to 5), the present study has provided examples of the use of TIs in research material. As Meehan (6) noted, there are different views as to whether all categories can be considered “real” TIs, or whether the lower categories function more as preparation and clarification. In this study, the five TI categories include two preparatory categories (1 and 2). Category 2 was frequently used. Category 3 was more demanding, as it encouraged the patient to reveal feelings and thoughts about what they believed the therapist thought about them. Categories 4 and 5 corresponded to interventions often understood as transference interpretations in a stricter sense (6); these categories were rare in the available clinical material.

In the extended examples from two adolescents, the therapists mainly used Category 1 and 2 TIs, only rarely Category 3, and no Category 4 or 5 TIs. The therapists often encouraged the patient to explore difficult feelings in relation to the therapist and other people outside of therapy. These findings invite discussion about differences in the use of TIs in therapeutic work with adolescents compared with adults. It may be more difficult to use more “proper” TIs with adolescents. This might reflect therapist-related explanations, and the lack of information about such influences represents a limitation in the study. In our material, it appears that even when therapists did not formulate a proper grammatical question, their interventions effectively ended in a question mark, typically conveyed through intonation. Whether this is specific to this group of therapists is unknown, but there may be cultural aspects to this rather humble way of offering interventions. Using Schwartz’s (28) cultural value orientations, Norway appears particularly high on egalitarianism and low on hierarchy, with strong norms of equality, flat structures, modesty, small power distance, and informality with authorities. In general, harmony is valued and open conflict is avoided. Interventions and interpretations presented as questions may represent a plausible microlevel enactment of these macrolevel cultural value orientations.

### 5.1 Limitations

The complexity underlying the results should be taken into consideration. When discussing and exploring the effects of TI in time-limited youth psychotherapy, it is necessary to be clear about what definition of TI is applied and to describe the technical use of TIs (frequency and level of transference intervention). Based on the present study, no conclusions can be drawn regarding whether these effects will last over time or what impacts TIs may have in other PDT-treatment modalities (e.g., longer treatments) or with other diagnostic groups. Our study includes a limited number of STPP therapies. Furthermore, the use of TI in youth psychotherapy may be culturally sensitive, and our examples may therefore not be representative of TIs in STPP in other countries. For future studies, youth-specific instruments such as MADRS for youth (MADRS-Y) should be considered (29).

## 6 Conclusion

There is a need to overcome barriers to individualized care and to integrate emerging evidence into practice. The findings may expand our understanding of the efficacy of Tis in adolescent depression and support the inclusion of transference work in the design of tailored therapeutic protocols for this vulnerable population.

In the clinical material examined from a study of STPP with adolescents experiencing depression, the therapists offered TIs to a moderate degree. In the present paper, the five categories of TIs and subsequent TWs were exemplified. Most of these interventions were preparatory (Categories 1–2), encouraging the patient to reveal thoughts and feelings about the therapy and the therapist. The material also showed less use of confrontation and traditional interventions (Categories 4–5).

## Data Availability

The datasets presented in this article are not readily available because the data are only accessible to the researchers. Requests to access the datasets should be directed to randiul@uio.no.
